# Interventions to enhance medication adherence in pregnancy- a systematic review

**DOI:** 10.1186/s12884-022-05218-5

**Published:** 2023-03-02

**Authors:** Anna Davies, Sadie Mullin, Sarah Chapman, Katie Barnard, Danya Bakhbakhi, Rachel Ion, Francesca Neuberger, Judith Standing, Abi Merriel, Abigail Fraser, Christy Burden

**Affiliations:** 1 Academic Women’s Health Unit, Translational Health Sciences, Bristol Medical School, University of Bristol, Bristol, BS8 1QU UK; 2 Department of Pharmacy and Pharmacology, University of Bath, Bath, BA2 7AY UK; 3 North Bristol NHS Trust, Southmead Hospital, Southmead Road, Bristol, BS10 5NB UK; 4 Population Health Sciences, Bristol Medical School, University of Bristol, Bristol, BS8 1QU UK; 5 National Institute for Health Research Bristol Biomedical Research Centre, University Hospitals Bristol and Weston NHS Foundation Trust and University of Bristol, Oakfield House, Oakfield Grove, Bristol, BS8 2BN UK

**Keywords:** Systematic review, Medication, Adherence, Maternal medicine, Perinatal outcomes, Chronic disease

## Abstract

**Background:**

Sub-optimal medication adherence in pregnant women with chronic disease and pregnancy-related indications has the potential to adversely affect maternal and perinatal outcomes. Adherence to appropriate medications is advocated during and when planning pregnancy to reduce risk of adverse perinatal outcomes relating to chronic disease and pregnancy-related indications. We aimed to systematically identify effective interventions to promote medication adherence in women who are pregnant or planning to conceive and impact on perinatal, maternal disease-related and adherence outcomes.

**Methods:**

Six bibliographic databases and two trial registries were searched from inception to 28th April 2022. We included quantitative studies evaluating medication adherence interventions in pregnant women and women planning pregnancy. Two reviewers selected studies and extracted data on study characteristics, outcomes, effectiveness, intervention description (TIDieR) and risk of bias (EPOC). Narrative synthesis was performed due to study population, intervention and outcome heterogeneity.

**Results:**

Of 5614 citations, 13 were included. Five were RCTs, and eight non-randomised comparative studies. Participants had asthma (*n* = 2), HIV (*n* = 6), inflammatory bowel disease (IBD; *n* = 2), diabetes (*n* = 2) and risk of pre-eclampsia (*n* = 1). Interventions included education +/− counselling, financial incentives, text messaging, action plans, structured discussion and psychosocial support. One RCT found an effect  of the tested intervention on self-reported antiretroviral adherence but not objective adherence. Clinical outcomes were not evaluated. Seven non-randomised comparative studies found an association between the tested intervention and at least one outcome of interest: four found an association between receiving the intervention and both improved clinical or perinatal outcomes and adherence in women with IBD, gestational diabetes mellitus (GDM), and asthma. One study in women with IBD reported an association between receiving the intervention and maternal outcomes but not for self-reported adherence. Two studies measured only adherence outcomes and reported an association between receiving the intervention and self-reported and/or objective adherence in women with HIV and risk of pre-eclampsia. All studies had high or unclear risk of bias. Intervention reporting was adequate for replication in two studies according to the TIDieR checklist.

**Conclusions:**

There is a need for high-quality RCTs reporting replicable interventions to evaluate medication adherence interventions in pregnant women and those planning pregnancy. These should assess both clinical and adherence outcomes.

**Supplementary Information:**

The online version contains supplementary material available at 10.1186/s12884-022-05218-5.

## Introduction

The number of pregnant women prescribed or recommended medication for chronic conditions is increasing [1]. A US study identified a 37% increase in pregnant women with chronic conditions including arthritis, inflammatory bowel disease (IBD), epilepsy and asthma between 2005 and 2014 [1]. In a European survey, 17% of pregnant women were taking medication for a chronic medical condition [2]. Pregnant women with chronic medical conditions, especially with multimorbidity, have greater risk of poorer perinatal outcomes including stillbirth, pre-term and caesarean delivery, maternal morbidity and mortality [3–7]. Additionally, an increasing proportion of women have pregnancy-related indications for medication such as gestational diabetes mellitus and hypertension, attributed to increasing maternal age and body mass index [8, 9].

For pregnant women with chronic diseases such as asthma [10], IBD [11, 12], rheumatological conditions [13], epilepsy [14] and those with pregnancy-related indications such as hypertension [15], appropriate medication adherence is associated with improved outcomes for mother and baby. Active disease has been associated with increased risk of pre-term birth in women with chronic medical conditions [16–18]. For conditions such as HIV it is crucial that medication adherence is optimal during pregnancy, when HIV transmission risk is greater, to achieve maximum viral load suppression and prevent mother to child transmission [19].

The UK National Institute for Clinical Excellence (NICE) recommends optimising medication use to control chronic disease before conception and throughout pregnancy for many (though not all) conditions where it is considered, on balance, safe and effective to take it [20]. However, many pregnant women do not adhere to their prescribed medicines. In a survey of Canadian women with IBD, 46% reported stopping at least one prescribed medication in pregnancy [21]. In a study across 18 countries, 36% of pregnant women with chronic conditions, including rheumatic, bowel and cardiovascular disorders reported low adherence. Adherence ranged between 17 and 56% according to condition [2]. In a study investigating adherence to low-dose aspirin prophylaxis to prevent pre-eclampsia in high-risk women, up to 46% of women reported non-adherence [22].

Interventions to support appropriate medication adherence in women planning pregnancy and pregnant women are therefore needed to improve maternal and fetal outcomes. Recent systematic reviews evaluating the evidence for interventions to increase adherence in the wider population have excluded pregnant women [23, 24]. Furthermore, the evidence was limited by risk of bias, and heterogeneity in intervention type, outcome assessed and measures used [23]. Interventions found to be effective in the general population may not be effective for pregnant and women planning pregnancy if they do not target pregnancy-specific adherence barriers, such as concerns about medication-related risks to the baby [25, 26].

## Objectives

We aimed to systematically identify effective interventions to support adherence to medications for chronic disease and pregnancy-related indications in pregnant women and women planning pregnancy and impact on perinatal, maternal disease-related and adherence outcomes.

## Methods

This report adheres to the PRISMA guidelines for systematic reviews of healthcare interventions [27, 28]. Patients were not involved in the development or conduct of this review.

### Funding

This grant for this review was awarded by the David Telling Charitable Trust (no reference provided) and was peer reviewed. The funder did not play a role in conducting this research or writing the paper.

### Registration

The protocol for this review was registered on the PROSPERO database (CRD42018104667, 25 July 2018).

### Eligibility criteria

We identified English-language, peer reviewed studies, meeting the following population, intervention, comparator, outcome, study design (PICOS) criteria [29]:

i) *Population****:*** Women who are pregnant or planning to conceive, prescribed or recommended regular, self-administered medication/s for any chronic disease or obstetric indication of any duration. We excluded studies reporting interventions where there is inconsistent evidence, resulting in lack of consensus that medication adherence improves clinical outcomes [30]. We excluded studies reporting interventions to support medication adherence to treat opioid addiction, smoking cessation and in mental health conditions; these conditions were outside the remit of this review.
ii) *Types of intervention:* Any intervention intended to promote medication adherence.
iii) *Control/Comparison groups****:*** those comparing an intervention with usual care, alternate intervention/s or before and after studies where intervention effects on outcomes were assessed over time.
iv) *Outcomes:*
  We searched the COMET database and CROWN initiative to identify relevant Core Outcome Sets(COS). We did not identify any COS relevant to studies of interventions to improve medication adherence in pregnant women or women planning to conceive.
  *We identified* studies assessing at least one of:
  - Perinatal outcomes for baby and mother: including but not limited to pre-term labour(< 37 weeks), birthweight, Apgar score [31], Neonatal Intensive Care Unit(NICU) admissions, maternal pre-eclampsia, perinatal death.
  - Maternal disease-related clinical outcomes up to 6 weeks postnatal**:** including but not limited to: measures of disease severity, activity or control(e.g. inflammatory markers, hospitalisations, intensive care admissions).
  - Objectively assessed measures of adherence in pregnancy (up to birth): including but not limited to refill data, pill count, pharmaceutical claims data, electronic monitoring, biological assay assessed during pregnancy or within 6 weeks of delivery to reflect adherence during the pregnancy.
  - Self-reported adherence in pregnancy (up to birth): self-report of missed/used doses, and validated questionnaires assessing non-adherence(e.g. Brief Medication Questionnaire [32]).
v) Study design: comparative experimental or observational study designs including randomised controlled trials, controlled trials, comparative studies, before and after studies, cohort studies, case-control studies.

### Information sources and search strategy

Search terms were developed by a specialist medical librarian. These included synonyms of medication, adherence, compliance, pregnancy, and conception (see Additional file 1 for strategy). The search was devised in Medline, with thesaurus terms adapted for other databases. The following MeSH terms were used: “medication adherence” “patient compliance”, “patient dropouts”, “pharmaceutical preparations”, and “pregnancy”.

Searches were conducted with no language restrictions and no limit on study design from database inception to 28th April 2022. The search was restricted to humans and adults and applied to six electronic databases and two clinical trials registries:

- Cochrane Central Register of Controlled Trials
- MEDLINE via Healthcare Databases Advanced Search(HDAS)
- Embase via HDAS
- CINAHL via HDAS
- British Nursing Index via HDAS
- PsycINFO via HDAS
- ClinicalTrials.gov [33]
- World Health Organization International Clinical Trials Registry [34]

### Study selection

We downloaded citations and screened them using Covidence [35]. Two authors screened all abstracts and full-texts of retained citations using the inclusion and exclusion criteria (see Table 1). Studies were allocated to an exclusion reason using the first criterion met. Disagreements were resolved through discussion with the senior authors. Where citations were abstracts for conferences or trial registrations or protocols, the reviewers made efforts to identify whether the study had been published in a peer-reviewed publication.

**Table 1:** Inclusion and exclusion criteria

Inclusion	Exclusion
• Written in English • Published, peer reviewed article • Reports primary data • Randomised controlled trial, controlled trial, observational study with a control group/condition, before and after study • Reports quantitative data on effectiveness of an intervention • Participants are pregnant women or women planning pregnancy • Intervention aims to increase adherence to prescribed medication for a chronic condition or pregnancy-related indication (e.g. pre-eclampsia risk) • Reports at least one of: perinatal, maternal clinical (< 6 weeks postnatal), objective or self-reported adherence to medication during pregnancy	• Not in English
	• Not a published, peer reviewed article
	• Not a full text article e.g. conference abstract, i.e. unlikely to provide adequate information to extract methodological and outcome data.
	• Does not report primary data (e.g. review, commentary, editorial), such that it does not give adequate data to assess of intervention effects on outcomes of interest.
	• Trial registration or protocol, i.e. no data available about study results
	• Effectiveness of an intervention not assessed e.g. descriptive study or qualitative assessment.
	• Studies without a control group or control timepoint.
	• Trial or observational study of an intervention with only one participant (e.g. case reports), thus are of limited validity due to high risk of bias
	• Does not report data about pregnant women or pre-conceptual women; reports data including both pregnant and non-pregnant sample that is not disaggregated.
	• Intervention does not aim to address medication adherence (e.g. addresses adherence to other parts of self-management such as blood glucose monitoring, physiotherapy)
	• Does not report at least one of perinatal, maternal clinical, objective or self-reported adherence outcomes during pregnancy
	• Trial ended or data withdrawn, thus data should not be included in review.
	• Trial aims to address medication adherence to opioid addiction treatment Opioid addiction adherence interventions may not be relevant to women with chronic conditions and pregnancy-related indications.
	• Trial aims to assess medication adherence to treatment in pregnant women with mental health conditions or smoking cessation. These studies are not relevant to the research objectives.
	• Trial aims to address adherence to iron supplementation for anaemia only- there is no clear evidence that adherence to medication for anaemia improves clinical outcomes.
	• Self-reported or objective adherence is not assessed during pregnancy.
	• Clinical outcomes are measured > 6 weeks postnatally and therefore is unlikely to reflect adherence during the pregnancy.

### Data extraction

For all data extraction (study data, intervention description and risk of bias), two authors extracted data independently for each study. Discrepancies were resolved through discussion.

#### Study data

We used a standardised, piloted form to extract data. Extracted data were: publication details, country, World Bank income group [36], health condition treated, population, number of participants, study design, intervention and control conditions, intervention details, outcomes assessed (categorised as perinatal outcomes for baby and mother, maternal disease-related clinical outcomes, objective and self-reported adherence, and intervention effectiveness (means and standard deviations, medians and IQR or counts/ percentages, odds ratios and 95% confidence intervals, and effect sizes where reported).

#### Adequacy of intervention description

A proforma based on the TIDieR checklist was used to extract data on adequacy of intervention descriptions [37]. This checklist examines in detail the replicability of the intervention by determining whether precise details of the intervention are provided. Extracted data were: intervention name; theory and evidence base for the intervention; what was the intervention; procedures used; intervention provider and skills/training received; how, where, when and frequency of delivery; tailoring reported; reported modifications; fidelity of delivery.

#### Risk of bias assessment

The Cochrane EPOC risk of bias tool for studies with a control group was used, and the tool for interrupted time series designs used for before and after studies [38].

### Data synthesis

There was methodological and clinical heterogeneity [39] across studies in conditions investigated, interventions, and clinical and adherence outcomes assessed. Therefore, it meta-analysis was not appropriate. A narrative summary is used to describe the evidence for each outcome type (perinatal, maternal clinical, objective and self-reported adherence), in relation to each health condition investigated.

## Results

### Study selection

**Fig. 1:**
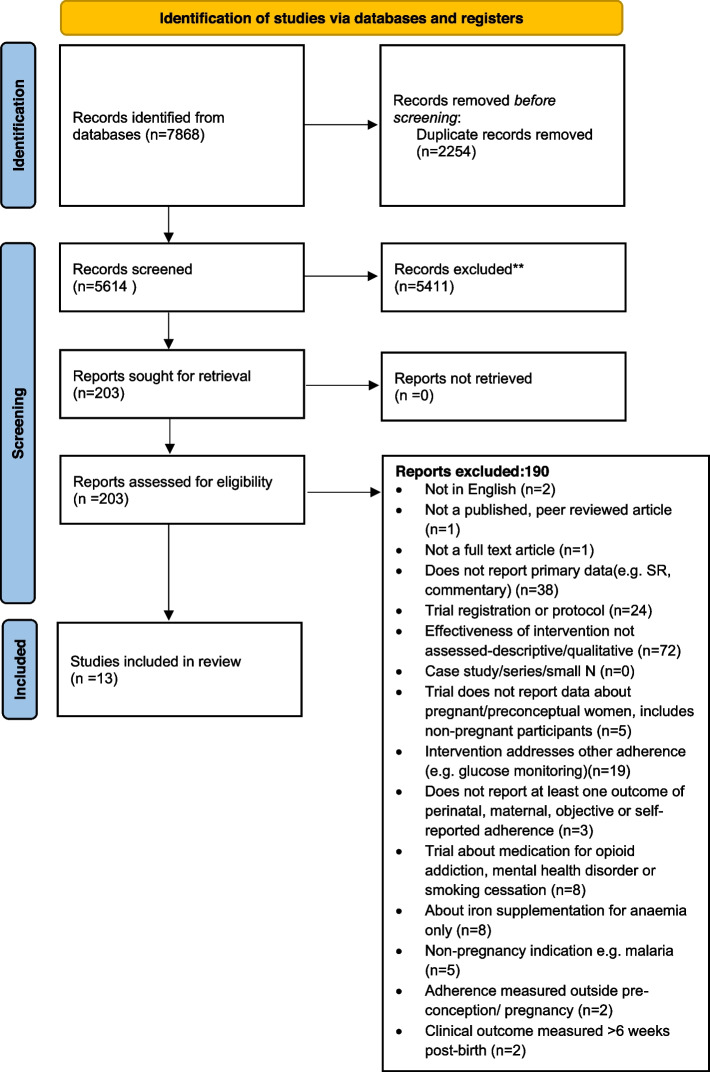
PRISMA diagram

The PRISMA flow diagram is provided in Fig. 1. Following de-duplication 5614 publications were screened. Thirteen studies met the inclusion criteria. Table 2 summarises study characteristics and findings.

**Table 2:** Study characteristics and perinatal, maternaland adherence outcomes

Citation & Country	Sample and Condition	Design	Total N analysed	Intervention (I)	Control/ comparison (C)	Perinatal and maternal clinical outcomes assessed	Results Intervention (I) vs Control/comparison (C)	Adherence outcomes assessed	Results I vs C
Baarnes et al., 2016 Denmark (HIC) [40]	Pregnant women with asthma	Before and after	*N* = 114	Verbal and written information about asthma treatment, importance of adherence with medication, at each appointment. Review every 4 weeks during pregnancy and 3 months after birth.	Pre-pregnancy or earlier gestation (at enrolment)	Control of asthma- per GINA guidelines Before measurement: enrolment; After measurement: last visit before birth.	Rated well controlled**: I:** 88% vs **C:** 66% (no inferential statistics reported).	Objective Adherence: MPR: pre-pregnancy to during pregnancy	All women: MPR: **I:** 46% (s.d. = 31) **C:** 28% (s.d. = 25; (*p* < .0001). Women who filled > 1 prescription: MPR pre-pregnancy 34% (s.d. = 24), pregnancy 56% (s.d. = 25), (*p* < .0001).
						Asthma control: (enrolment to last visit before birth).	Improvement in FEV_1_ (p < .05), Reduction in FeNO (p < .001)	SRA: rated by woman as good, moderate, low (enrolment to last visit).	‘Good’ adherence**; I:** 73%, **C:** 52%, (*p* > .001)
Carter et al., 2020 USA (HIC) [41]	Pregnant women with Type 2 (48.7%) and gestational diabetes (GDM) diagnosed < 32 weeks (51.3%)	Pilot RCT	*N* = 78 (I = 48;, C = 30)	4 Group education sessions: Self-assessed + recorded blood pressure, weight blood glucose. Activities: snacks, self-reflection activities, crafts, pregnancy or behavioural health stations + group activities about diabetes, pregnancy, tailored behavioural health topics. Seen individually as needed.	Individual care: prenatal care in diabetes clinic every 2 weeks or more at provider discretion per national guideline. Included routine screening, review of blood sugar logs, medication titration.	Perinatal outcomes	IoL: **I:**52.5%, **C:**42.1%, *p* = 0.36; C-section: **I:** 50%, **C:** 52.6%, *p* = 0.82; GAB: **I:** 37.8 (s.d. = 3.1), **C:** 37.5 (s.d. = 2.2), *p* = 0.75; PTB **I:** 20%, **C:** 23.7, *p* = 0.69; BW: **I:**3256.1 g (s.d. = 817.5), **C:**3176.7 g (s.d. = 644.2), *p* = 0.64; SmGA: **I:**5.0%, **C:**2.6%, *p* > 0.99; LGA: **I:** 42.5%, **C:**28.9%, *p* = 0.21; DYS: **I:**12.5%, **C:**2.6%, *p* = 0.20; Neonatal polycythemia: **I:** 10%, **C:** 15.8%, *p* = 0.51; Hypoglycaemia: **I:** 27.5%, **C:** 26.3%, *p* = 0.91; Treatment for hypoglycaemia: **I:** 17.5%, **C:** 15.8%, *p* = 0.84; Respiratory distress syndrome: **I:** 12.5%, **C:** 21.1%, *p* = 0.37; NICU admission> 24 hrs: **I:** 27.5%, **C:** 26.3%, p = 0.91	SRA: Summary of self-care diabetes activities(no. of days taking recommended medication in past week)	I = 6.4 days (s.d = 1.5); C: 5.7 days (s.d. = 2.4) *p* = 0.48
						Maternal: HbA1c (type 2 group only), Hypertensive disorders (both groups). % and mmol/mol	HbA1c: **I:** 6.3% (s.d. = 0.7%) (44.9 mmol/mol; s.d. = 7.8 mmol/mo) l, **C:** 6.8% (s.d. = 0.9%) (50.7 mmol/mol, s.d. = 10.0 mmol/mol), *P* = 0.09 Hypertensive disorder: **I:** 40% **C:** 34.2%, *p* = 0.34;		
De Lima et al., 2016 Netherlands (HIC) [42]	Women planning pregnancy within 2 years and pregnant women with Inflammatory Bowel Disease	Comparative (non-randomised)	*N* = 317 (I = 155; C = 162)	Pre-conception counselling (PCC) in specialist clinic: discussed guidelines, letter with advice summarised. Followed up every 3 months until conception, 2 months during pregnancy (2-weekly if active disease).	Attending specialist clinic during pregnancy only. (Follow-up every 2 months during pregnancy only, 2 weekly if active disease)	Perinatal outcomes	LBW: **I:** I7.2% vs **C:** 12.6% (aOR 0.08; 95% CI, 0.01–0.48) *p* < .006 BW: **I** 3373 g(IQR295–3679), **C**: 3363 g (IQR 2829–3630) p=.52 SmGA: **I:** 31%, **C**: 9.4% (aOR 0.22; 95% CI, 0.05–1.00), p=.05 GAB: **I:** 38.4wks (IQR 34.0–40.0), **C:** 38.0 wks (36.1–39.5), *p* = .50 LB: **I:** 75.2%, **C:** 78.4% (aOR 0.79; 95% CI 0.45–1.38), p=.40 PTB: **I**: 13.4%, **C:** 7.9% (aOR 1.74; 95% CI 0.73–4.6), p=.22 SA: **I:** 20.2%, **C:** 19.1% (aOR 1.10; 95%CI 0.61–2.00), p=.75 CA: **I:** 3.1%, **C:** 4.7% (aOR 1.74; 95% CI 0.73–4.6), p=.91	SRA: ‘Correct adherence’- measure used not reported	Not adherent: **I**: 2.6%, **C**:13.6%; Adherent: **I**: 97.4%, **C:** 86.4% (aOR, 5.69; 95% CI 1.88–17.27, *p* < .002).
						Maternal disease activity: (HBI; SCCAI; OR fecal calprotectin >200μg/g).	Disease activity: **I:** 18.1%, vs **C**:34.0%; No disease activity: **I**: 58.1%, **C:** 63.0% (aOR, 0.51; 95% CI, 0.28–0.95, *p* = .05)		
Flannagan et al., 2020 Australia (HIC) [43]	Pregnant women and women planning to conceive with IBD (50% pregnant; median gestation 12 weeks)	Before and after	*N* = 81	Single-session gastroenterologist led in person/ telephone using pro-forma for evidence based advice, structured discussion relating to IBD and pregnancy + information tailored to patient concerns.	Before attending intervention (timepoint not stated)	Maternal disease activity: IBD questionnaire (bowel symptoms and systemic symptoms subscales)	Bowel symptoms **I:** 5.5 (IQR 4.9–6.1), **C:** 6 (IQR 5.5–6.5), *P* < .001 Systemic symptoms: **I:** 4.4 (IQR 3.4–5), **C:** 4.8 (IQR 4.2–5.6), *p* < .01	SRA: medication adherence scale, (not reported).	80% doses: **I:**93%, **C:** 89%; 50–80% doses: **I:** 5%, **C:**4%; 0–49% doses **I:** 7%, **C:** 1%; *p* = 0.18
Karunia et al., 2019 Indonesia (LMIC) [44]	Pregnant women with high risk for pre-eclampsia (PE) screened 11 + 0 to 13 + 6 weeks of pregnancy	Before and after	*N* = 12	Educational booklet and verbal information given twice 28 days apart (visit 1 and 2): definitions, signs, symptoms and effects of PE, prevention with low dose aspirin, dosing and administration, info about benefits of aspirin for mother and foetus, importance of adherence.	After first intervention administration			Objective: Pill count (pills remaining from 30 given) at 28 days post second intervention administration	**I:** 95.8% (third visit), **C:** 89.8% (second visit), *p* = 0.011
Kim et al., 2019 Malawi (LIC) [45]	Pregnant women with HIV	Pilot RCT	*N* = 30 (I = 146 C = 160)	VITAL Start (VS): Video and counselling intervention, based on IMB Model: information about ART on body, managing side effects, adherence strategies.	Health care worker delivered 1 hr. group lecture based on Malawi National standard pre-ART counselling flipchart.			Objective adherence: Electronic pill count derived from pharmacy records at 1 month	Pill count > 90–100%: **I**: 65.1%, **C:** 59.4%, *p* = .31.
								SRA: missed dose	Missed dose last 7 days: **I:** 13.6%, **C:**26.8%, *p* < .02. Missed dose last 30 days: **I:** 29.9% **C:** 15.3%, *p* < .02.
Krishnakumar et al., 2020 India (LIC) [46]	Pregnant women with gestational diabetes mellitus using metformin or insulin (44 using metformin, 37 insulin)	Before and after	*N* = 81	Patient education leaflet and verbal education for 30 minutes. Two sessions. Referred to as continuous.	Pre-intervention measurement (timepoint not stated)	Maternal: Glycaemic Control- Fasting and post-prandial glucose (mg/dL) pre-intervention to 2/3 months follow-up.	Fasting: Insulin group: **I:** 94.59 (s.d.5.77), **C:** 103.81 (s.d. = 7.98), *p* = .0001; Metformin group: **I:** 94.84 (s.d. = 6.18), **C:** 105.16 (s.d. = 15.16),, *p* < .0001; Postprandial blood glucose: Insulin group: **I:** 116.05 (s.d. = 6.01), **C:**128.30 (s.d. = 7.26), *p* < .0001; Metformin group: **I:** 117.86 (s.d. = 6.54), **C:** 130.23 (s.d. = 16.83), *p* < .0001	SRA: Morisky medication adherence scale	MMAS (whole sample, *n* = 81) **I:** 6.38 (s.d. = 0.70 **C:** 5.6 (s.d. = 1.15), *p* < .0001; Metformin group: I 6.43 (s.d.0.36), **C:** 4.84 (s.d. = 1.14)*p* < .0001, Insulin (*n* = 37): **I:** 6.62 (s.d. = 0.53),C: 4.77 (s.d. = 1.17), *p* < .0001
Murphy et al., 2005 Australia (HIC) [47]	Pregnant women with asthma	Before and after	*N* = 177	Asthma education programme +/− action plan, offered two visits, with additional visits where required.	First visit (approx. 20 weeks gestation).	Perinatal outcomes: (comparing women +/− action plan, *n* = 46). Before measurement: first visit or 20wks gestation. After measurement: 33 wks gestation.	Lower BW in women without additional action plan (no data reported; *p* < .005).	SRA: ICS adherence in the previous week at 20 vs 33 weeks. Severe asthma (*n* = 61)	All participants: Adherence > 80% doses**. I:** 40%, **C:** 21% (*p* < .006) Severe asthma: Decreased non-adherence between measurements points (exact values not reported; *p* = 0.014)
						Lung function: Measured 20 weeks gestation vs.33 weeks in women with mild (max *n* = 108), moderate (max *n* = 42) and severe asthma (max *n* = 61)	*Mild and moderate asthma:* No change between visits (*p* > .05 for all): FEV_1_L; FEV_1_ (% pred); FVC-L; FEC_1_:FVC; night symptoms; morning symptoms; activity limitation; RMU: d/w, t/d, t/w. *Severe asthma:* No change between visits (*p* < .05) for FEV_1_L; FEV_1_ (% pred); FVC-L; FEC_1_:FVC; morning symptoms; activity limitation.^a^ Night symptoms: **I:** 0 (IQR0–3), **C:** 5 (IQR 2–7), *p* < .05 RMU-d/w: **I:** 7 (IQR 2–7) **C:** 7 (IQR 7–7), *p* < .05 RMU-t/d: **I:** 2 (IQR 1–3) **C:** 3 (IQR 2–5), *p* < .05 RMU-t/w: **I:** 7(IQR 3–14), **C:** 21 (IQR 7–35), *p* < .05		
Pintye et al., 2020 Kenya (LMIC) [48]	Pregnant women with HIV	Comparative (non-randomised)	*N* = 356 (I = 190; C = 166)	2-way SMS message communication (mWACH-PrEP); weekly automated messages informed by behaviour change theory + opportunity to communicate with a nurse via SMS	Women starting PrEP in preceding month before implementation of mWACH-PrEP			SRA: Self-reported adherence- number of missed doses in the past month in those returning, (high = < 1 missed pill/week)	High PrEP adherence **I:** 73% **C:** 55% (aRR = 1.35; 95% CI = 1.28,1.41; *p* < .001).
								Objective: PrEP refills; PrEP continuation (attendance + PrEP Refill)	I: 43%, C: 22%; aRR = 1.75; 95% CI 1.21, 2.55; *p* = .003
Potter et al., 2019 USA (HIC) [49]	Pregnant women with HIV	Comparative (non-randomised)	*N* = 117 (I = 14 C = 103)	Centering pregnancy; group sessions on ARVs in pregnancy, importance of adherence and impact, preventing transmission. (10 × 2 hours)	Standard care	Maternal viral load near birth (< 200 copies/mL).	**I** vs **C**: (aOR 7.0; 95% CI 0.6–81.51, p.13)		
						Perinatal outcomes	BM [aOR 0.29; 95% CI 0.07–1.10], APGAR at 5 mins [*p* > .99; OR not reported], GAB [aOR 0.29, 95% CI, 0.07–1.10], BW [*p* = .51, OR not reported], Newborn HIV status: [*p* = .73, OR not reported].		
Psaros et al., 2022 South Africa (UMIC) [50]	Pregnant women with HIV	RCT	*N* = 23	Individual-targeted combined depression and adherence intervention: problem solving. Therapy and LifeSteps for PMTCT.	Usual care			SRA: Adherence composite score 1 month adherence, 3 month follow up.	No main effect or interaction between intervention and timepoint: BL: **I:** 85.3 (s.d. = 12.9), **C:** 79.0 (s.d. = 14.4), Post-test: **I:** 92.8 (s.d. = 8.7), **C:** 77.8 (s.d. = 15.4), 3 month follow up: **I:** 88.7 (s.d. = 9.3), **C:** 89.2 (s.d. = 5.9).
								Objective: Medication event monitoring system (bottle with digitised cap)	No main effect or interaction between intervention and time point. BL: **I:** 99.4 (s.d. = 2.2), **C:** 92.9 (s.d. = 14.3), Post test: **I:** 98.6 (s.d. = 3.0), **C:** 100 (s.d. = 0); 3 month follow upFollow up: **I:** 92.8 (s.d. = 6.4), **C:** 85.7 (s.d. = n/a)
Weiss et al. 2014 South Africa (UMIC) [51]	Pregnant women with HIV	Pilot RCT (cluster randomised trial)	*N* = 478 (I = 238, C = 240)	PartnerPlus: comprehensive couples-based PMTCT programme: 4, weekly sessions included CBT to improve adherence to treatment. Informed by IMB Model.	Enhanced standard care: standard antenatal care + PMTCT, health-related videos, without PartnerPlus intervention.	Perinatal outcomes: LB in subset (n = 82)	LB**: I:** 81.1%, vs **C:** 86.0% (*p* = .49)	Objective adherence: blood sample assessing ARV presence in mother (n = 24) and infant (n = 25)	No difference between groups for ARV presence in blood Mother **I:** 75%**, C:** 50% (*p* = .19) **I**nfant **I:** 92%, **C:** 75% (*p* = .32)
Yotebieng et al., 2016 D.R. Congo (LIC) [52]	Pregnant women diagnosed with HIV, seropositive. newly diagnosed, < 32 weeks pregnant	RCT	*N* = 326 (I = 171, Cl = 155)	Compensation ($5, plus $1 increment at each subsequent visit) conditional on attending scheduled clinic visits and accepting offered PMTCT services.	Standard care	Maternal viral load at 6 weeks postpartum (detection: 40 copies/mL)	Detectable **I:** 33.9% vs **C:** 30.3% [aRisk difference = −.01, 95% CI −0.10-.08).	Objective adherence: pill count assessed and classified as 100% adherent.	Intervention: adherent **I:** 69.9%, **C:** 68.1%; Not adherent **I:** 30.1%, **C:** 31.9% [aRisk difference = 0.03, 95%CI − 0.05- 0.12]

*LIC* lower income country, *HIC* higher income country, *UMIC* upper middle income country, *I* Intervention Group, *C* Control/comparison, *FeNo* Exhaled nitric oxide, *FEV*_*1*_ Forced expiratory volume, *FEC* forced expiratory capacity, *FVC* forced vital capacity, *FVC-L* FCV-Liters, *HBI* Harvey Bradshaw Index, *SCCAI* Simple Clinical Colitis Activity Index, *ARVs* antiretroviral medication, *PMTCT* prevention of mother-to-child transmission, *PrEP* Pre-exposure prophylaxis, *CBT* Cognitive Behavioural Therapy, *IMB* Information Motivation Behavioural Skills Model, *IoL* Induction of Labour, *DYS* Shoulder dystocia, *BW* birth weight, *LBW* low birth weight (< 2500 g), *LB* live births, *SmGA* small for gestational age (below 10th centile), *LGA* Large for gestational age (>90^th^centile), *GAB* gestational age at birth (weeks), *PTB* pre-term births (< 37 weeks), *SA* spontaneous abortions, *MC* miscarriage, *CA* congenital abnormalities, *BM* birth mode, *PC* Pill Count, *RMU (t/d; d/w; t/w)* Reliever medication use (times/day; days/week; times/week), *LMP* last menstrual period. ^a^ see Additional file 3 for full dataset

### Characteristics of included studies

#### Design, participants and conditions investigated

Five studies were RCTs [41, 45, 50–52], three were comparative studies in which women were not randomised to their group [42, 48, 49], and five used a before and after design [40, 43, 44, 46, 47]. Five studies were carried out in Lower and Middle Income Countries (LMIC) and Low Income Countries(LICs) [44–46, 48, 52], and eight in High Income Countries (HICs) and Upper Middle Income Countries(UMICs) [40–43, 47, 49–51]. Conditions investigated were asthma (*n* = 2 [40, 47]), inflammatory bowel disease (IBD; *n* = 2 [42, 43]), Human Immunodeficiency Virus (HIV; *n* = 6 [45, 48–52]) women with type 2 and gestational diabetes mellitus (T2DM, GDM; *n* = 2 [41, 46]), and women with risk of pre-eclampsia (*n* = 1 [44]). In eleven studies the participants were pregnant women [40, 45, 47, 49, 51, 52], and two studies delivered an intervention prior to, and during pregnancy [42, 43].

#### Nature of interventions and quality of intervention reporting

Interventions included individual or group education with and without written materials [40–42, 44, 46, 49], and education alongside counselling [45], written action plans [47], problem solving and LifeSteps counselling to treat depression and adherence [50], psychosocial support and cognitive behavioral skills [51], two-way SMS messaging [48], structured discussion and tailored advice with treating clinician [43], and one provided cash transfers to incentivise adherence [52]. Interventions were delivered to individuals [40, 42–44, 47, 48, 50, 52], and groups [41, 45, 49, 51]. In one study it was unclear whether it was a group or individual intervention [46]. In five studies the intervention was delivered face-to-face [41, 43, 45, 51, 52], and five combined face-to-face delivery with written information [40, 42, 44, 47, 49]. In two studies the mode of delivery was unclear [46, 50]. The intervention was delivered on multiple occasions in eleven studies [40–42, 44, 46–52], and on a single occasion in two studies [43, 45].

**Fig. 2:**
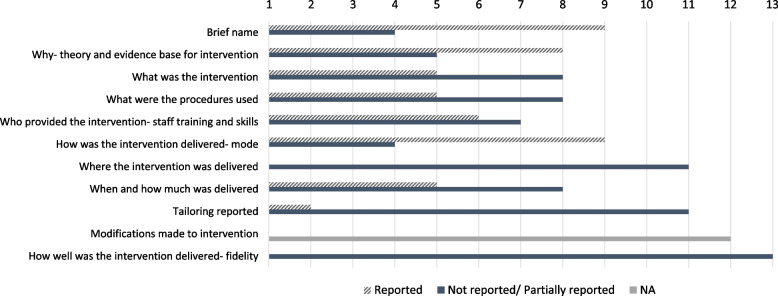
Quality of intervention reporting based on the TIDieR criteria

Two studies provided sufficient detail about their intervention to enable accurate replication [43, 48]. The remaining studies provided limited information to enable replication according to the TIDieR checklist (Fig. 2 and Additional file 2).

### Risk of Bias of included studies

Tables 3 and 4 present the risk of bias assessment for each study. Twelve studies were at high risk of bias in at least one domain [40–50, 52], with one study showing low or unclear risk of bias [51].

**Table 3:** Assessment of risk of bias for studies with a control group (EPOC)

Study ID	Study design	Random sequence generation	Allocation concealment	Blinding of participants and personnel	Blinding of outcome assessment	Incomplete outcome data	Selective reporting	Baseline outcome measurements similar	Baseline Characteristics similar	Knowledge of allocated interventions adequately prevented	Protection against contamination	Other bias
Carter et al., 2020 [41]	RCT	Low	Low	High	Unclear	Low	Low	High	Low	High	High	
de Lima et al., 2016 [42]	Comparative (non-randomised)	High	High	Unclear	Unclear	Low	High	Unclear	High	Unclear	Low	Selection
Kim et al., 2019 [45]	RCT	Unclear	Unclear	Unclear	Unclear	High	Low	Unclear	Low	Unclear	High	
Potter et al., 2019 [49]	Comparative (non-randomised)	High	High	High	Unclear	Low	Low	Low	High	Unclear	High	Selection; unbalanced group sizes
Pintye et al., 2020 [48]	Comparative (non-randomised)	High	High	High	High	High	Low	Unclear	Unclear	High	Low	
Psaros et al., 2022 [50]	RCT	Low	Unclear	Unclear	High	High	Low	High	Low	Unclear	Unclear	Small sample
Weiss et al., 2014 [51]	RCT	Unclear	Unclear	Unclear	Unclear	Low	Low	Low	Low	Unclear	Low	
Yotebieng et al., 2016 [52]	RCT	Unclear	unclear	Unclear	Unclear	High	Low	Low	Low	Unclear	Low	Loss to follow up no ITT.

**Table 4:** Assessment of risk of bias for studies without a control group

Study ID	Study design	Intervention independent of other changes	Intervention unlikely to affect data collection	Knowledge of the allocated interventions adequately prevented during the study	Incomplete outcome data affecting result	Selective outcome reporting	Other of bias
Baarnes et al., 2016 [40]	Before and After	High	Low	High	Low	Low	High
Flannagan et al., 2021 [43]	Before and After	High	Low	High	Low	Low	Low
Karunia et al., 2019 [44]	Before and after	High	Low	High	Low	Low	High
Krishnakumar et al., 2020 [46]	Before and after	High	Low	High	Low	Low	High
Murphy et al., 2005 [47]	Before and After	High	Low	High	Low	Low	High

### Synthesis of results

Eight studies assessed both clinical outcomes (perinatal and/or maternal) and adherence (self-report or objective) [40–43, 46, 47, 51, 52]. One study explored perinatal and maternal clinical outcomes only [49], and four studies self-reported and/or objective adherence only [44, 45, 48, 50].

### Perinatal outcomes

Studies investigating perinatal outcomes included one RCT in women with HIV [51], one RCT in women with GDM and T2DM [41], two non-randomised comparative studies in women with IBD and HIV [42, 49], and a  before and after study in women with asthma [47]. Heterogeneous outcomes were assessed (Table 1).

#### HIV

An RCT compared the effect of a *PartnerPlus* couples-based intervention including education and cognitive-behavioural skills for pregnant women with HIV in the South Africa. In a sub-sample of women (*n* = 82), compared with usual care, no difference in percentage of live births was found [51].

In a non-randomised comparative study, pregnant women with HIV in the USA selected to attend either *CenteringPregnancy* group education sessions or usual prenatal care (*n* = 117). No differences were found for all outcomes: caesarean/ vaginal births, Apgar score at five minutes, gestational age at birth, birth weight or newborn HIV status [49].

#### Diabetes

 A pilot RCT in the USA that compared a group-based educational intervention with normal, individual care in women with GDM and T2DM, reported no impact of the intervention on induction of labour, caesarean sections, gestational age at birth, pre-term birth, birth weight, small for gestational age(<10th centile), large for gestational age (> 90th centile), shoulder dystocia, neonatal polycythemia, hypoglycaemia and treatment for hypgolycaemia, respiratory distress and NICU admission > 24 hours [41].

#### Inflammatory Bowel Disease(IBD)

 In a non-randomised comparative study in women with IBD (*n* = 317), the effect of receiving guideline-informed education and care in a specialist clinic in women who attended prior to conception and through pregnancy, was compared with the effect of receiving it during pregnancy only [42]. Those attending the clinic prior to conception were less likely to have a low birth weight baby (< 2500 g) compared with those who did not [42]. The authors found no associations between group and birth weight, small for gestational age baby, gestational age at birth, live births, spontaneous abortions, preterm birth and congenital abnormalities.

#### Asthma

 In a before and after study in women with Asthma in Australia [47], sub-analysis to investigate the impact of receiving or not receiving a written asthma action plan was conducted (n not reported). Women with a written action plan had higher birthweight babies than those that did not. 

### Maternal clinical outcomes

Eight studies reported on maternal clinical outcomes [40–43, 46, 47, 49, 52] (see Table 1).

#### HIV

Two studies investigated an intervention’s effect on maternal viral load in women with HIV at different time points: near birth [49], and six weeks post-partum [52]. In an RCT conducted in the Democratic Republic of Congo (*n* = 326), financial incentives to attend a clinic and acceptance of prevention of mother to child transmission of HIV treatment (PMTCT) were tested. There was no difference in maternal viral load at 6 weeks post-partum between intervention and control participants [52]. In a non-randomised comparative study conducted in the USA (*n* = 117), *CenteringPregnancy* group education compared with standard care was not associated with maternal viral load at birth [49].

#### Asthma

Two before and after studies investigated control of asthma [40, 47]. A close-monitoring and education intervention delivered during pregnancy was associated with improved asthma control in the close monitoring period (last visit prior to birth) compared with prior to or in early pregnancy (before intervention) [40].

An asthma education programme was not associated with differences in maternal asthma control between early (20 weeks’ gestation) and late gestation (33 weeks’ gestation) in women with mild and moderate asthma (FEV_1_, FEV_1_-L, FEV_1_:FVC), night and morning symptoms, activity limitation and reliever medication use. In women with severe asthma, however, the education programme was associated with improved night-time symptoms and reliever medication use (reflecting reduced exacerbations) relating to days per week, times per day and times per week at 33 weeks’ gestation [47].

#### IBD

Two studies explored the impact of an adherence intervention in pregnant women and women planning to conceive with IBD [42, 43]. In a non-randomised comparative study, guideline informed specialist care prior to conception and during pregnancy, versus during pregnancy only, was associated with less disease activity during pregnancy (*n* = 317; Harvey Bradshaw Index; Simple Clinical Colitis Activity Index; faecal calprotectin) [42]. In a before and after study in Australia (*n* = 81), in a sample of women who were pregnant or planning pregnancy, evidence-based, structured discussion and information tailored to patient needs reduced bowel and systemic symptoms between the pre- and post-intervention measurements, assessed using subscales of an IBD questionnaire [43].

#### Diabetes

Two studies explored the impact of an intervention on glycaemic control in women with GDM and T2DM [41, 46]. In a pilot RCT (*n* = 78) in the USA, women with GDM and T2DM received a group-based educational intervention or usual, individual care. The authors reported no effect of group education on HbA1c in women with T2DM (antental HbA1c was not measured in the women with GDM) [41]. In a before and after study of women with GDM using metformin or insulin (*n* = 81), providing a patient education leaflet and verbal education across two sessions improved fasting and post-prandial blood glucose for both women taking insulin and women taking metformin at 2–3 months’ follow-up, compared with a pre-intervention measurement [46].

### Objective medication adherence

Seven studies assessed objective medication adherence using heterogenous measures [40, 44, 45, 48, 50–52].

#### Asthma

A close monitoring and regular review intervention tested in a before and after study in Danish women (*n* = 114), found increased medication possession ratio (reflecting increased adherence) during the pregnancy (last visit before birth) than in the period prior to intervention (pre- or early pregnancy). In sub-analyses, adherence in women who filled at least one prescription at enrolment was higher during than before pregnancy [40].

#### HIV

Four RCTs, and a non-randomised comparative study investigated the impact of an intervention on objective adherence measures in pregnant women with HIV [45, 48, 50–52]. There was no effect of the *PartnerPlus* programme on presence of antiretroviral medication in the mothers’ or infants’ blood [51]. In two studies there was no effect on antiretroviral pill count for cash incentives [52], or the VITAL Start video-based intervention [45]. In a small RCT in South Africa (*n* = 23), the authors reported no effect of a problem solving therapy and a LifeSteps intervention compared with usual care on adherence, measured using a Medication Event Monitoring System at two follow-up measurements [50]. In a non-randomised comparative study in Kenya (*n* = 256), the adherence of women receiving a 2-way SMS messaging intervention with personalised messages (mWACh-PrEP) was compared with that of women receiving standard antenatal care before implementation of mWACh-PrEP. The investigators reported increased adherence, assessed as medication refill and clinic attendance in women who received mWACh-PrEP [48].

#### Pre-eclampsia risk

In a small before and after study (*n* = 12) of pregnant women at increased pre-eclampsia risk, aspirin adherence was measured using pill count on two occasions: at 28 days after first administration of an information booklet about pre-eclampsia, and 28 days after the second administration, thus assessing the impact of one versus two doses of the intervention. Authors reported an increase in adherence between first and second administration of the intervention [44].

### Self-reported medication adherence

Nine studies reported data on self-reported medication adherence [40–43, 45–48, 50]. One study described assessing it in their method but did not report outcome data for adherence to antiretroviral medication [51].

#### Asthma

Two before and after studies assessed self-reported adherence in women with asthma. In a study investigating a close-monitoring intervention (*n* = 114), self-reported adherence reported in an interview was rated by the woman as good, moderate or low. Adherence increased during the close-monitoring period (measured last visit before birth) compared with adherence at enrolment into the study (early/ pre-pregnancy) [40]. For an asthma education programme (*n* = 177), clinicians asked women about adherence to inhaled corticosteroids [47]. Non-adherence (taking fewer than 80% of doses) decreased following the intervention between the pre-intervention early gestation visit (20 weeks’ gestation) and late gestation (approximately 33 weeks’ gestation). In a sub-analysis of women with severe asthma there was decreased non-adherence between early and late gestation.

#### HIV

Three studies reported data on self-reported adherence in an HIV PMTCT intervention programme. In a pilot RCT in Malawi the VITAL start video intervention was compared with lecture-style counselling (*n* = 306) [45]. VITAL start participants had a lower number of self-reported missed doses in the preceding seven and 30 days. In a small RCT in South Africa (*n* = 23), there was no increase in self-reported adherence at post treatment or 3-month follow up for receiving problem solving therapy and a LifeSteps intervention compared with usual care [50]. A non-randomised comparative study in Kenya (*n* = 356), reported increased self-reported adherence (number of missed doses) among pregnant women receiving mWACh-PrEP (2-way SMS intervention), compared with women who had received usual care prior to implementation of mWACh-PrEP [48].

#### IBD

In a non-randomised comparative study in the Netherlands (*n* = 317), women with IBD receiving guideline informed care prior to conception and during pregnancy compared with during pregnancy only was associated with better self-reported adherence (tool not described) to IBD medication during pregnancy [42]. In a before and after study in Australia (*n* = 100), pregnant women and women planning pregnancy received tailored evidence based advice and structured discussion around pregnancy. The authors reported no increase in adherence at follow-up compared with measurements taken before administration of the intervention [43].

#### Diabetes

In a pilot RCT in the USA (*n* = 78) comparing group education with usual, individual care in women with GDM and T2DM, there was no difference in the number of days women took their recommended medication, assessed using a validated scale [41]. In a before and after study in India (*n* = 81) a patient education leaflet and verbal education for women taking metformin or insulin was associated with increased self-reported adherence at 2–3 months’ follow-up compared with before administration of the intervention, using a validated adherence tool [46].

## Discussion

We aimed to identify effective interventions to support medication adherence in pregnant women and women planning pregnancy. We have identified a dearth of high-quality RCTs evaluating interventions to support medication adherence in pregnant women with chronic conditions and pregnancy-related indications. This review identified 13 studies assessing the effectiveness of interventions to improve medication adherence on perinatal, and maternal disease-related clinical outcomes, and/or adherence. Only five RCTs met our inclusion criteria; all were rated as having unclear or high risk of bias. Only one study aimed to improve aspirin adherence for a pregnancy-related indication (prophylaxis for pre-eclampsia).

Within the available evidence eight studies reported findings suggestive of an effect of their tested intervention on at least one outcome of interest. Of these, only one was an  RCT. A video intervention based on the Information Motivation Behavioral Skills model [53] was effective for self-reported adherence to antiretroviral therapy in women with HIV, but not for observed adherence, assessed as pill count [45]. This study did not assess clinical maternal or perinatal outcomes. The interventions tested in the seven studies employing non-randomised comparative, and before and after designs warrant further investigation in appropriately designed RCTs.

Our findings are consistent with a previous review of medication adherence interventions in non-pregnant populations [23]. We identified considerable heterogeneity in the interventions tested, outcomes assessed and measures used, preventing a meta-analysis; we could not group interventions by type due to heterogeneity in the types of interventions delivered (counselling, videos, incentives, text messaging, lifestyle intervention, specialist clinics). Several studies used inadequate sample sizes to assess intervention effectiveness. There was also inadequate reporting of interventions tested. While many described their intervention in broad terms, only two studies provided adequate detail about all aspects of the intervention to enable accurate replication of the tested intervention according to the TIDieR checklist [37]. The CONSORT statement recommends adequate reporting of intervention content to allow replication and evidence synthesis [54]. Intervention reporting guidelines should be used in future intervention studies to achieve this [37], and should include reference to the specific behavior change techniques employed to enable their future use [55].

Of the 13 included studies, eight reported both adherence and clinical (perinatal/maternal) outcomes, and one reported on clinical outcomes only. Of note, a before and after study [43] reported that the intervention was associated with improved clinical outcomes but not medication adherence. Several explanations are plausible, including greater power to detect differences in continuous versus categorical outcomes (clinical scores vs adherence), confounding in the case of non-randomised studies, and an effect on clinical outcomes via mechanisms other than medication adherence, such as improvement in other health-related behaviours. Formal mediation analyses were not undertaken. While improvements in clinical outcomes are the primary aim of intervening to improve medication adherence, future RCTs should assess both adherence measures and clinical outcomes, to explore further the underlying mechanisms that lead to improved pregnancy outcomes. Included studies assessed perinatal outcomes less frequently than maternal clinical outcomes and self-reported or objectively assessed adherence. Adherence measures are not an adequate proxy for improved perinatal outcomes, since objective measures may assess only recent adherence, and self-report may be impacted by social desirability biases [56]. An understanding of the impact of interventions that improve adherence and maternal clinical outcomes on perinatal outcomes is needed to ensure they will achieve this important clinical benefit. A further limitation of the current literature is that different measures were used to assess the same outcomes across studies within the same healthcare domain. For example, studies assessing intervention effects for pregnant women with asthma used different measures to assess lung function. Similarly, of nine studies measuring self-reported adherence, three used a validated tool, however the selected tool differed across studies. To support further evidence synthesis, consistent outcome reporting across trials for women with the same condition or pregnancy-related indication will be beneficial. A standardised set of outcomes to be assessed across all pregnancy medication adherence studies may be beneficial. The Core Outcome Set currently under development by the OMERACT initiative may be relevant [57].

A Cochrane review of adherence interventions in the wider population identified that adherence intervention effects are typically modest [23], and that more effective interventions are needed. Interventions informed by behavioural theory and/or evidence about barriers to adherence are more likely to be effective than those based on intuition [26]. Within the current review, eight studies reported using theory or evidence to develop their intervention. Previous studies have identified factors that may influence medication adherence in pregnancy including sociodemographic characteristics [2] and beliefs about medications such as concerns or decreased belief that medication is beneficial or necessary [58]. In addition, women with chronic illness report receiving inconsistent information about medicines during pregnancy and breastfeeding [59]. Both women and staff may have concerns about medication use in pregnancy, and may perceive a need to balance risks to the baby with benefits to the mother of reducing active disease. Many may believe that non-adherence to medications is safer than the perceived risks of taking it, due to limited evidence about their safe use in pregnancy [25, 26].

Inadequate medication adherence is associated with increased morbidity, mortality, and health care costs in the non-pregnant population. A recent systematic review has highlighted that despite the evidence that medication non-adherence places a significant cost burden on healthcare systems, research assessing the economic impact of medication non-adherence is limited and of varying quality [60]. The health-related consequences of non-adherence for both chronic and obstetric conditions in pregnancy include uncontrolled disease and complications such as pre-term delivery, impacting maternal and infant health outcomes in both the short and long term. Therefore, cost impact for non-adherence to medication could be substantial and should be considered in future research in this population. It is therefore important to identify effective interventions to support appropriate medication adherence.

### Strengths and limitations

The strengths of this review are the use of a registered, pre-specified protocol, and a systematic, reliable process using multiple databases to identify relevant studies, from database inception to the present day. Study selection criteria and data extraction were reliably applied and conducted by two researchers. We used an inclusive approach to identify as much relevant data as possible, including data from RCTs and non-randomised study designs, and used established checklists to assess the risk of bias and quality of intervention reporting. A potential limitation of this review is that we may not have identified all relevant studies; this may have resulted from poorly indexed literature in the field of medication adherence [23]. An additional limitation of this review was that it was not possible to conduct meta-analysis due to identified clinical and methodological heterogeneity of included studies.

### Implications for practice, policy and public health

The findings of this review indicate that there is currently only limited and low-quality evidence for the use of any of the tested interventions in clinical care. The rapidly increasing number of women prescribed or recommended medication for chronic disease and pregnancy-related indications, and increasing evidence of sub-optimal medication adherence in pregnant women, suggests that policymakers should seek to facilitate research efforts in this area, for example, through prioritisation or tailored funding calls. This is particularly important given the potential healthcare-related costs and clinical burden of poorly managed disease in this population.

## Conclusion

Only 13 studies, the majority of which are of poor methodological quality, have assessed interventions to improve medication adherence. Effective interventions, evaluated in high-quality RCTs are needed. Interventions should be replicable, informed by theory and evidence, and studies should assess their effect on clinically meaningful, as well as economic outcomes.

## Supplementary Information

**Additional file 1.** Search strategy (medline). Medline search strategy.

**Additional file 2.** TIDieR Extraction data. TIDieR data for each study.

**Additional file 3.** Additional data Murphy et al. Additional data extracted from Murphy et al. study.

## Data Availability

The dataset used and analysed during the current study are available from the corresponding author on reasonable request.

## Abbreviations

TIDieR: Template for intervention description and replication
EPOC: Effective practice and organization of care
RCT: Randomised controlled trial
HIV: Human immunodeficiency virus
IBD: Inflammatory bowel disease
US: United States
NICE: National Institute for Health and Care Excellence
PRISMA: Preferred Reporting Items for Systematic Reviews and Meta-Analyses
PROSPERO: International prospective register of systematic reviews
PICOS: Population, intervention, control, outcome, setting
COMET: Core outcome measures in effectiveness trials
CROWN: Core outcomes in women’s and newborn health
NICU: Neonatal intensive care unit
MeSH: Medical subject headings
LMIC: Lower middle income country
LIC: Lower income country
HIC: High income country
UMIC: Upper middle income country
T2DM: Type 2 diabetes mellitus
GDM: Gestational diabetes mellitus
HbA1c: Haemoglobin A1c
OMERACT: Outcome Measures in Rheumatoid Arthritis Clinical Trials
