# Supplementary material for: Interventions to enhance medication adherence in pregnancy- a systematic review
Source: BMC Pregnancy Childbirth. 2023 Mar 2;23:135. doi: 10.1186/s12884-022-05218-5 (PMC9979410; doi:10.1186/s12884-022-05218-5)
Supplement: Supplementary file 1 — Additional file 1. Search strategy (medline). Medline search strategy. [file 12884_2022_5218_MOESM1_ESM.docx]

Additional file 1: Search strategy (Medline)

| 1 | ((Medication* OR drug* OR regimen* OR biologic* OR treatment*) ADJ3 (adher* OR compliance OR comply OR non-compliance OR noncomplicance OR concordance OR cooperat* OR co-operat*)).ti,ab |
| --- | --- |
| 2 | "MEDICATION ADHERENCE"/ |
| 3 | (1 OR 2) |
| 4 | (Patient ADJ (compliance OR adher* OR non-compliance OR noncompliance OR cooperat* OR co-operat* OR dropout*)).ti,ab |
| 5 | "PATIENT COMPLIANCE"/ |
| 6 | "PATIENT DROPOUTS"/ |
| 7 | (4 OR 5 OR 6) |
| 8 | (medication* OR drug*).ti,ab |
| 9 | exp "PHARMACEUTICAL PREPARATIONS"/ |
| 10 | (8 OR 9) |
| 11 | (7 AND 10) |
| 12 | (3 OR 11) |
| 13 | (Pregnan*).ti,ab |
| 14 | (gravidit*).ti,ab |
| 15 | (Gestation*).ti,ab |
| 16 | exp PREGNANCY/ |
| 17 | (13 OR 14 OR 15 OR 16) |
| 18 | (12 AND 17) |
| 19 | exp ANIMALS/ NOT HUMANS/ |
| 20 | (exp CHILD/ OR exp INFANT/ OR exp ADOLESCENT/) NOT exp ADULT/ |
| 21 | (19 OR 20) |
| 22 | 18 not 21 |

*The thesaurus terms were adapted for the other databases*
