# Supplementary material for: Interventions to enhance medication adherence in pregnancy- a systematic review
Source: BMC Pregnancy Childbirth. 2023 Mar 2;23:135. doi: 10.1186/s12884-022-05218-5 (PMC9979410; doi:10.1186/s12884-022-05218-5)
Supplement: Supplementary file 2 — Additional file 2. TIDieR Extraction data. TIDieR data for each study. [file 12884_2022_5218_MOESM2_ESM.docx]

**Additional file 2: TIDieR extraction data**

| **Authors** | **1. Brief Name Y/N** | **2. WHY: study** describes any rationale/ theory/ goal of the elements essential to the intervention (e.g. how intervention maps to identified barriers from literature or patient group, theory used) | **3. WHAT:** Describes physical or informational materials including those for participants, used in intervention delivery or in training of providers. (Yes, No, partially, NA) | **4. PROCEDURES** Describes the procedures, activities, and/or processes in the intervention, including any enabling/support activties (Yes, No, Partially NA) | **5. WHO PROVIDED**: Describes who delivered it e.g. midwife, nurse, psychologist, their background expertise (Yes, No, NA) | **6. HOW:** mode of delivery described (e.g. face to face, internet, telephone, mixture) (Yes, No partially, NA) | **7. WHERE:** describes locations, including any relevant infrastructure or features (e.g. large room?? Setting with IT access? Local health centre).(Yes, No, partially, NA) | **8. WHEN AND HOW MUCH:** Describes when sessions were delivered, how frequently, number of times per month, length of sessions, over period of what time? (partially= some elements described)(Yes, No, partially, NA) | **9. TAILORING:** if the session (or part of it) was tailored to the individual or adapted, describe what, when, how, why (Yes, No, partially, NA) | **10. MODIFICA-TIONS:** was the intervention changed during the study. If yes, how? (y= modifications described, n= modifications made but not described how, NA= no modifications reported) | **11. HOW WELL:** Did they assess intervention adherence or fidelity? (Yes, No, partially, NA) |
| --- | --- | --- | --- | --- | --- | --- | --- | --- | --- | --- | --- |
| Baarnes et al 2016 | Y | N | P | P | Y | Y | P | P | P | NA | N |
| Carter et al., 2020 | Y | Y | P | Y | Y | Y | P | Y | P | NA | N |
| de Lima 2016 | Y | P | N | P | N | Y | P | P | N | NA | N |
| Flannagan et al., 2021 | Y | Y | Y | Y | Y | Y | Y | Y | Y | NA | N |
| Karunia et al., 2019 | N | Y | Y | P | P | Y | N | Y | N | NA | N |
| Kim et al., 2019 | Y | Y | Y | P | P | P | P | N | P | NA | N |
| Krishnakumar et al., 2020 | N | Y | P | N | N | N | N | P | N | NA | N |
| Pintye et al., 2000 | Y | Y | Y | Y | Y | Y | NA | Y | Y | NA | N |
| Psaros et al., 2022 | Y | Y | Y | Y | Y | N | N | P | P | Y | P |
| Murphy et al., 2005 | N | P | P | P | P | Y | N | Y | P | NA | N |
| Potter et al., 2019 | Y | Y | P | Y | Y | Y | P | P | N | NA | N |
| Weiss et al., 2014 | Y | P | N | P | P | Y | P | P | N | NA | N |
| Yotebeing et al., 2016 | N | P | P | N | N | N | N | P | N | NA | N |
| YES | **9** | **8** | **5** | **5** | **6** | **9** | **1** | **5** | **2** | **1** | **0** |
| No/ Partial | **4** | **5** | **8** | **8** | **7** | **4** | **11** | **8** | **11** | **0** | **13** |
| **NA** | **0** | **0** | **0** | **0** | **0** | **0** | **0** | **0** | **0** | **13** | **0** |
