# Supplementary material for: Interventions to enhance medication adherence in pregnancy- a systematic review
Source: BMC Pregnancy Childbirth. 2023 Mar 2;23:135. doi: 10.1186/s12884-022-05218-5 (PMC9979410; doi:10.1186/s12884-022-05218-5)
Supplement: Supplementary file 3 — Additional file 3. Additional data Murphy et al. Additional data extracted from Murphy et al. study. [file 12884_2022_5218_MOESM3_ESM.docx]

Additional file 3: Additional data Murphy et al., 2005

| **Mild asthma** (max n=108) No changes between visits (p>.05 for all)   1. **FEV_1_L: I:** 3.13 (se=0.08), **C:** 3.14 (s.e.=0.05) 2. **FEV_1_ (% pred):** **I:** 102 (s.e.=2), **C:** 102 (s.e.=2) 3. **FVC:-L:** **I:** 3.83 (s.e.=0.09), **C:** 3.79 (s.e.=0.06) 4. **FEV_1-_L:** **I:** 2.91 (s.e.=0.12), **C:** 2.87(s.e.=0.08) 5. **FEV_1_: FVC: I:** 0.82 (s.e.=.01), **C:** 0.83 (s.e=.01) 6. **Night symptoms:** **I:** 0 (IQR 0-0). **C:** 0 (IQR 0-0) 7. **Morning symptoms:** **I:** 0 (IQR 0-0), **C:** 0 (IQR 0-0) 8. **Activity limitation: I:** 0 (IQR 0-0), **C:** 0 (IQR 0-0), 9. **Reliever use days/wk:** **I:** 0 (IQR 0-3), **C:** 0 (IQR 0-2), 10. **Reliever use times/day:** **I:** 0 (IQR 0-1), **C:** 0 (IQR 0-1) 11. **Reliever use times/wk:** **I:** 0 (IQR 0-3), **C:** 0 (IQR 0-2)   **Moderate asthma (max n=42)** No changes between visits (p>.05 for all)   1. **FEV_1_ L:** **I:** 2.91 (s.e.=0.12), **C:** 2.87(s.e.=0.08) 2. **FEV_1_ (% pred):** **I:** 96 (3), **C:** 96 (3), 3. **FVC-L: I:** 3.80 (s.e.=0.11), **C:** 3.61 (s.e.=0.07) 4. **FEC_1_:FVC: I:** 0.76 (s.e.=.02), **C:** 0.80 (s.e=.02), 5. **Night symptoms:** **I:** 0 (IQR 0-1), **C:** 0 (IQR 0-2) 6. **Morning symptoms:** **I:** 0 (IQR 0-2), **C:** 1 (IQR 0-7) 7. **Activity limitation:** **I:** 0 (IQR 0-1), **C:** 0 (IQR 0-4) 8. **Reliever use days/wk:** **I:** 7 (IQR 1-7), **C:** 7 (IQR 2-7) 9. **Reliever use times/day: I:** 2 (IQR 1-2), **C:** 2 (IQR 1-3) 10. **Reliever use times/wk:** **I:** 7 (IQR 1-14), **C:** 12 (IQR 2-21)   **Severe asthma (max n=61)** No change between visits (p>.05) for:   1. **FEV_1_ L:** **I:** 2.91 (s.e.=0.12), **C:** 2.87(s.e.=0.08) 2. **FEV_1_ (% pred):**  **I:** 96 (s.e.=3), **C:** 95 (s.e.=3) 3. **FVC-L: I:** 3.80 (s.e.=0.11), **C:** 3.61 (s.e.=0.07) 4. **FEV_1_:FVC: I:** 0.76 (s.e.=.02), **C:** 0.80 (s.e=.02) 5. **Morning symptoms: I:** 2 (IQR 0-7), **C:** 4 (IQR 0-7) 6. **Activity limitation:** **I:** 0 (IQR 0-6), **C:** 1 (0-5)   FEV_1_= Forced expiratory volume; FEC= forced expiratory capacity; FVC= forced vital capacity; FVC-L=FCV-Liters |
| --- |
